# Evidence for no sexual isolation between *Drosophila albomicans* and *D. nasuta*

**DOI:** 10.1002/ece3.619

**Published:** 2013-06-03

**Authors:** Yong-Kyu Kim, Dennis R Phillips, Yun Tao

**Affiliations:** 1 Department of Biology, Emory UniversityAtlanta, Georgia, 30322; 2 Department of Chemistry, University of GeorgiaAthens, Georgia, 30602; 3 Howard Hughes Medical Institute, Janelia Farm Research Campus19700 Helix Drive, Ashburn, Virginia, 20147

**Keywords:** Courtship, cuticular hydrocarbons, *D. albomicans*, *D. nasuta*, mating behavior, speciation

## Abstract

Sexual isolation, the reduced tendency to mate, is one of the reproductive barriers that prevent gene flow between different species. Various species-specific signals during courtship contribute to sexual isolation between species. *Drosophila albomicans* and *D. nasuta* are closely related species of the *nasuta* subgroup within the *Drosophila immigrans* group and are distributed in allopatry. We analyzed mating behavior and courtship as well as cuticular hydrocarbon profiles within and between species. Here, we report that these two species randomly mated with each other. We did not observe any sexual isolation between species or between strains within species by multiple-choice tests. Significant difference in the courtship index was detected between these two species, but males and females of both species showed no discrimination against heterospecific partners. Significant quantitative variations in cuticular hydrocarbons between these two species were also found, but the cuticular hydrocarbons appear to play a negligible role in both courtship and sexual isolation between these two species. In contrast to the evident postzygotic isolation, the lack of sexual isolation between these two species suggests that the evolution of premating isolation may lag behind that of the intergenomic incompatibility, which might be driven by intragenomic conflicts.

## Introduction

Species are usually “defined” by reproductive isolating mechanisms that maintain them as separate gene pools (Dobzhansky 1937; Mayr 1942). There are a number of ways to prevent gene flow between different species (reviewed in Coyne and Orr 2004). When two species meet, one or both species may refuse to mate the other species (premating isolation); when mating does occur in nature or is forced to occur by experimenters, fertilization can be difficult (postmating and prezygotic isolation), or the resulting offspring will often be inviable or sterile (postzygotic isolation). In the genus *Drosophila*, allopatric species pairs generally evolve premating and postzygotic isolation at similar rates, whereas premating isolation evolves much faster than postzygotic isolation in sympatric species because direct selection might be involved in evolving premating isolation through reinforcement (Coyne and Orr 1989, 1997).

One of the premating isolation mechanisms is sexual isolation, where the ability of one species to mate successfully with the other is reduced. Sexual isolation has been observed between many allopatric species, even though natural selection has never been able to act directly on this trait. Therefore, sexual isolation may often be an unintended result or byproduct of natural selection working on traits under sexual selection within each species (Andersson 1994), but apparently sexual isolation is not itself the target of natural selection. Before actual mating happens, a male and a female often need to determine the genetic qualities of mating partners by exchanging multiple signals, including acoustic, chemical, and visual cues. As a consequence of sexual selection (Andersson 1994), many species possess species-specific mating rituals and cues even among closely related species. These rituals and cues are often used by individuals to distinguish conspecific from heterospecific mates (reviewed in Markow and O'Grady 2005). One commonly used cue is the cuticular hydrocarbons (CHCs) that have been known to play pheromonal roles in *Drosophila* mate recognition and courtship (reviewed in Ferveur 2005). There is significant intra- and interspecific variation in CHCs among many species, and CHCs are part of the architecture of reproductive isolation between geographic strains and between species (Etges and Jackson 2001; Liimatainen and Jallon 2007; Alves et al. 2010; Kim et al. 2012; Sharma et al. 2012). In recent years, advances in molecular and statistical tools have facilitated the elucidation of the genetic bases for traits influencing sexual isolation between species (Doi et al. 2001; Takahashi et al. 2001; Ting et al. 2001; Shaw and Parsons 2002; Gleason and Ritchie 2004; Moehring and Mackay 2004; Gleason et al. 2009).

*Drosophila nasuta* (Duda 1924) and *D. albomicans* (Lamb 1914) belong to the *nasuta* subgroup of the *D. immigrans* species group and are distributed allopatrically (Wilson et al. 1969). *Drosophila nasuta* is found from East Africa through the Seychelles Islands and Mauritius to Sri Lanka and Peninsular India, whereas *D. albomicans* is distributed from Japan through Southern China and Indochina to the eastern states of India (Kitagawa et al. 1982). The two species are morphologically similar, but differ karyotypically. *Drosophila nasuta* (2*n* = 8) retains the ancestral karyotype while *D. albomicans* (2*n* = 6) has the derived fusions of the sex and the third chromosomes (Ranganath and Hägele 1981). Molecular data place the divergence time between these two species at ∼120,000 years ago (Bachtrog 2006). While the X is conserved across the genus *Drosophila* (Muller's A element), the 2L and 2R chromosome arms of these two species correspond to Muller's B and E elements, respectively, and the third chromosome consists of Muller's C and D elements (Chang et al. 2008). F_1_ hybrids between the two species can be easily produced in the laboratory. Interspecific F_1_ hybrids are apparently fertile (Kitagawa et al. 1982; Chang and Ayala 1989), but F_1_ hybrid males between *D. albomicans* females, especially Japanese strains, and *D. nasuta* males produced F_2_ offspring with a female-biased sex ratio (Chang and Ayala 1989; Inoue and Kitagawa 1990; Ohsako et al. 1994). Hybrid breakdown is also commonly observed in F_2_ and F_3_ generations of the crosses between these two species (Inoue and Kitagawa 1990). This sex ratio distortion is the result of sex chromosome meiotic drive (Yang et al. 2004), a phenomenon in which viable X- and Y-bearing gametes are transmitted unequally to offspring (Sandler and Novitski 1957).

Sex ratio meiotic drive is a common phenomenon often observed in well-studied taxa such as *Drosophila* (Jaenike 2001). Evolutionary arguments posit that sex ratio distortion and its subsequent suppression might have many evolutionary ramifications including epigenetic regulation of sex chromosomes and speciation (Meiklejohn and Tao 2010). It may also influence the evolution of sexual behaviors, as demonstrated in *D. pseudoobscura* where sex ratio distortion encourages the evolution of polyandry in that species (Price et al. 2008).

There are a few studies investigating sexual isolation between *D. nasuta* and *D. albomicans* (Kitagawa et al. 1982; Ramachandra and Ranganath 1987; Tanuja et al. 2001a; Chang and Tai 2007), but the conclusions are somewhat inconsistent. Kitagawa et al. (1982) reported random mating, whereas others observed significant sexual isolation (Ramachandra and Ranganath 1987; Tanuja et al. 2001a; Chang and Tai 2007). In addition, Chang and Tai (2007) reported asymmetrical sexual isolation in which more homogamic matings occurred in *D. albomicans* than in *D. nasuta*. Such inconsistency in the previous work warrants a reexamination of premating isolation between these two species. Additionally, it would be interesting to infer whether evolution of sex ratio distortion in *D. albomicans* has left any mark on the sexual behavior of this species.

Here, we analyze mating behavior and courtship as well as CHC profiles within and between *D. nasuta* and *D. albomicans*, using four geographic strains each. Observation of matings and statistical methods are employed to detect whether there is sexual isolation between these two species. The roles of courtship and CHCs in intraspecific and interspecific mating are analyzed. The results of our mating observations are compared with earlier studies on sexual isolation between these two species.

## Methods

### *Drosophila* stocks and handling

Three stocks of *D. nasuta* were obtained from the *Drosophila* Species Stock Center at San Diego, CA: 14030-1781.00 (Mysore, India), 14030-1781.06 (Mombasa, Kenya), and 14030-1781.13 (Cameroon). One *D. nasuta* strain (G86, Mauritius) was received from Masayoshi Watada, Ehime University, Japan, along with four other *D. albomicans* strains: E-10802 (MYH01-5, Miyakojima, Japan), E-10806 (IR96-13, Iriomotejima, Japan), E-10811 (KM01-5, Kumejima, Japan), and E-10815 (SHL48, Shilong, India). Hereafter we use the abbreviations Mys, Mom, Cam, Mau, MYH, IR, KM, and SHL to represent these 8 strains.

In these experiments, all *Drosophila* cultures were kept at room temperature (*ca*. 22 ± 1°C) in 13-dram plastic vials on a standard food medium made of cornmeal, yeast, molasses, and agar with propionic acid and Tegosept as mold inhibitors. Virgin males and females were collected every 8 h. To reduce the effects of density on the mating behavior of adults, a maximum of 20 virgin flies of the same sex were kept in each holding vial, where they were aged for 5 days before experimentation.

### Observations of matings

We observed matings in “multiple choice” tests where 12 pairs of males and females from each of two strains were placed in chambers as described earlier (Elens and Wattiaux 1964). We used four strains from each of the two species to set up pairwise tests, with a total of 16 interspecific and 12 intraspecific combinations. These observation chambers were circular, ∼12 cm in diameter and 1.3 cm deep, with a transparent plastic top and filter paper across the bottom. The bottom of the chamber was coated with a thin layer of fly instant medium (Carolina Biological cat. HB-173200; Burlington, NC). We introduced all 48 flies in a trial through a hole into the chamber using an aspirator and without anesthetization. The subject flies were 5-day-old virgins, and their strain identities were distinguished by notching the wings of only one strain, with notching alternated between the two strains in replicate trials. Four replicate chambers were observed for each combination of strains. In these two species, wing notching did not affect male activity during courtship or mating, nor was notching correlated with female discrimination of mates (data not shown). All matings were recorded for 60 min at room temperature. Females mated only once during our observations, and copulations lasted ∼25 min. Following the established protocol (Casares et al. 1998, 2005), we limited statistical analyses to the first 12 matings if more than 12 matings occurred within 1 h because matings would be underestimated if flies become less available after 50% individuals of the population had already mated (Casares et al. 1998).

### Statistical analyses of mating behavior

The joint isolation index (Malogolowkin-Cohen et al. 1965) was commonly used to measure the degree of sexual isolation between two strains, but it has been criticized for not being statistically robust due to uncorrected marginal effects (Merrell 1950; Gilbert and Starmer 1985; Rolán-Alvarez and Caballero 2000). Rolán-Alvarez and Caballero (2000) defined a new index, *I*_PSI_, using a pair sexual isolation (PSI) statistic that estimates mate choice coefficients for each type of mating pair. For each mating pair, PSI is calculated as following:


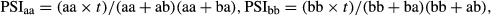



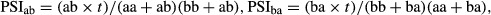


where *t* = aa + ab + ba + bb, the total number of matings. The numbers of aa, bb, ab, and ba represent the counts of matings between females and males of strain A, females and males of strain B, females of strain A and males of strain B, and females of strain B and males of strain A, respectively. The PSI statistics for all mating pairs are then used to obtain a new index (*I*_PSI_) as below:


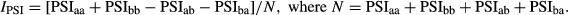


The index *I*_PSI_ considerably reduces the statistical bias of other indices including the joint isolation index, but retains the advantage of a simple relationship with the frequencies of homogamic and heterogamic matings (Pérez-Figueroa et al. 2005). Its significance can be obtained by permutation, as implemented in the software JMATING (Carvajal-Rodriguez and Rolán-Alvarez 2006).

We also calculated mating propensity coefficients (*W*) for both sexes using raw data from mating observations. The *W* statistic measures relative tendency to mate between two types of individuals of the same sex. For example, 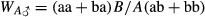, where *A* and *B* are the number of male *A* and *B* in a trial, respectively, and aa, bb, ab, and ba represent the number of matings as defined above. By definition, 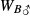 is the reciprocal of 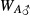. The significance of each *W* tested against the null hypothesis of equal mating success between the two types of individuals of the same sex can be obtained by permutation implemented in the JMating software (Carvajal-Rodriguez and Rolán-Alvarez 2006).

### Courtship

We observed courtship behavior between pairs of male and female from the same 8 strains of *D. albomicans* and *D. nasuta* used in the mating experiments, following the no-choice test format (Kim and Ehrman 1998). A total of 32 intraspecific and 32 interspecific combinations were observed, with 10 replicates for each combination. For each trial, a pair consisting of a 5-day-old male and female was introduced without anesthetization into a small Elens-Wattiaux chamber (2.5 cm in diameter). The bottom of the chamber was coated with a thin layer of fly instant medium (Carolina Biological cat. HB-173200). The activity of the insects was recorded until copulation occurred or for 10 min, whichever was shorter, using a JVC video camera (JVC Electronic Inc., Wayne, NJ) and a SONY monitor (SONY Electronics Inc., San Diego, CA). The time spent performing each individual element of courtship behavior was recorded along with courtship latency (CL, time elapsing between the male and females being put together and the male beginning first courtship), courtship duration (total time spent on each and all courtships from the first courtship to copulation or until the 10-min observation ended), and copulation latency (CPL, time elapsing from introduction to copulation). The courtship index (CI, the percentage of courtship duration over CPL, or 10 min if no mating occurred) was subsequently calculated. The CL was Log_10_ transformed and the Arcsine transformation was applied to the CI to improve normality of the data. Means were compared by analysis of variance (ANOVA).

### CHCs analysis

We quantified the CHCs of both sexes from each of four strains of *D. albomicans* and *D. nasuta* with five replicates, following previous procedure (Kim et al. 2004). After virgin flies were collected, flies were individually aged for 5 days in vials containing regular food. Note that CHC profiles are affected by age, mating status, and food as well as sexual interaction (Everaerts et al. 2010). Individual flies were placed in 1-mL cylindrical glass vials with Teflon caps. After 5 min, the flies were agitated for 2 min at a low speed using a vortex mixer and then removed from the extract. The extract was slowly evaporated to dryness under nitrogen and stored at −20°C until analysis, when it was allowed to warm to room temperature. After adding 20 μL of hexane, the vial was agitated for 2 min before 5 μL of the extract was injected into a Hewlett-Packard 5890 Series II gas chromatography (GC), equipped with an HP 5971A mass selective detector (MSD, quadruple mass spectrometer, Hewlett Packard, Palo Alto, CA). The GC was programmed to start at 170°C, with an increase of 2°C per min until 280°C. Ten peaks were identified to individual hydrocarbon molecules with the aid of the Wiley registry (7th edition) of mass spectral data, NIST 98 spectra for Agilent Chemstation (Hewlett Packard), and hydrocarbon standards. Two additional peaks at retention times (min), 54.90 and 58.80, were not included for data analysis because they were not consistently detected across the samples and quantities were very small. The HP Chemstation RTE integrator (version B.02.05) was used with default parameters to integrate the peaks, which was then normalized by dividing the total quantity of all 10 hydrocarbons. Thus, the peak areas were expressed as proportional values and were subsequently Log_10_ transformed to improve their normality. We performed a principal component (PC) analysis on the data set with JMP® V9.0 (SAS Institute Inc., Cary, NC), and the PCs with eigenvalues >1 were further analyzed.

## Results

### Sexual isolation among geographic strains of *D. albomicans* and *D. nasuta*

Using multiple-choice tests in the Elens-Wattiaux chamber, we observed homogamic and heterogamic matings in six intraspecific combinations using four geographic strains for each of the two species as well as 16 interspecific combinations among these strains. Four replicate chambers were run for each combination. The null hypothesis of random mating between the strains was first tested by a 2 × 2 contingency *χ*^2^ test, and then measured by *I*_PSI_, the isolation index, by using the PSI coefficients. The mating propensity coefficients (*W*) for each of the four types of flies in a combination were also calculated. All these results are summarized in Table 1. There is only one combination (Mau × Cam, both of *D. nasuta*) showing nonrandom mating among all 12 intraspecific and 16 interspecific combinations by the *χ*^2^ test, and it is only with marginal significance (*P* = 0.05). After Bonferroni correction made on multiple tests (*n* = 28), the significance disappeared. The same combination was flagged by *I*_PSI_*,* also with marginal significance (*P* = 0.05). We did not detect any significant interactions between the two species on sexual isolation indices across mating combinations (ANOVA, *P* < 0.5319).

**Table 1:** Sexual isolation and sexual selection between intraspecific and interspecific strains of *Drosophila albomicans* and *D*. *nasuta*

Strain A × Strain B	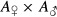	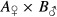	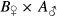	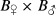	Random mating	Sexual isolation	Sexual selection			
		
					*χ*^2^	*I*_PSI_	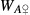	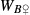	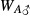	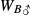
Intraspecific combination										
MYH × KM	8	18	4	18	1.01	0.18	1.18	0.85	0.33	3.00***
MYH × IR	11	12	8	17	1.26	0.17	0.92	1.09	0.66	1.52
MYH × SHL	15	15	8	10	0.14	0.06	1.67	0.60	0.92	1.09
KM × IR	14	6	17	11	0.44	0.10	0.71	1.40	1.82	0.55*
KM × SHL	17	10	10	11	1.13	0.16	1.29	0.78	1.29	0.78
IR × SHL	12	8	15	13	0.20	0.07	0.71	1.40	1.29	0.78
Mau × Cam	2	15	12	19	3.86*	−0.35*	0.55	1.82*	0.41	2.43**
Mau × Mom	8	21	3	16	0.90	0.18	1.53	0.66	0.30	3.36***
Mau × Mys	4	16	3	25	0.81	0.18	0.71	1.40	0.17	5.86***
Cam × Mom	14	12	10	12	0.34	0.09	1.18	0.85	1.00	1.00
Cam × Mys	19	13	8	8	0.38	0.10	2.00	0.50*	1.29	0.80
Mom × Mys	7	11	10	20	0.15	0.06	0.60	1.67	0.55	1.82*
Interspecific combination										
MYH × Mau	17	8	11	12	2.00	0.21	1.09	0.92	1.40	0.72
KM × Mau	23	5	17	3	0.07	−0.06	1.40	0.71	5.00	0.20***
IR × Mau	22	6	14	6	0.46	0.11	1.40	0.71	3.00	0.33***
SHL × Mau	19	8	16	5	0.20	−0.08	1.29	0.78	2.69	0.37***
MYH × Cam	5	22	3	18	0.15	0.08	1.29	0.78	0.20	5.00***
KM × Cam	18	8	13	9	0.54	0.11	1.18	0.85	1.82	0.55*
IR × Cam	15	11	8	14	2.17	0.22	1.18	0.85	0.92	1.09
SHL × Cam	14	11	8	14	1.81	0.20	1.14	0.88	0.88	1.14
MYH × Mom	18	14	10	6	0.17	−0.07	2.00	0.50*	1.40	0.71
KM × Mom	22	7	10	9	2.79	0.26	1.53	0.66	2.00	0.50*
IR × Mom	14	11	15	8	0.43	−0.10	1.09	0.92	1.53	0.66
SHL × Mom	11	14	15	8	2.17	−0.22	1.09	0.92	1.18	0.85
MYH × Mys	12	17	5	14	1.14	0.17	1.53	0.66	0.55	1.82*
KM × Mys	16	12	14	6	0.82	−0.14	1.40	0.71	1.67	0.60
IR × Mys	12	10	10	16	1.24	0.17	0.85	1.18	0.85	1.18
SHL × Mys	9	18	9	12	0.46	−0.10	1.29	0.78	0.60	1.67

MYH, Miyakojima; KM, Kumejima; IR, Iriomotejima; SHL, Shilong; Cam, Cameroon; Mau, Mauritius; Mom, Mombasa; Mys, Mysore. A 2 × 2 chi-square test was performed to test significant departure from random mating. The isolation indices measure the degree and direction of sexual isolation: *I*_PSI_, Isolation index with PSI coefficients (Carvajal-Rodriguez and Rolán-Alvarez 2006). *W*, relative mating propensity coefficient, estimates the effect of sexual selection in a mating.

* *P* < 0.05;

** *P* < 0.01;

*** *P* < 0.001.

Mating propensity for each type of flies in the multiple-choice tests, on the other hand, varied across the mating combinations. Females showed significant differences in mating propensity in 2 of the 12 intraspecific mating combinations (Mau × Cam, and Cam × Mys), whereas males showed significant differences in six combinations (MYH × KM, KM × IR, Mau × Cam, Mau × Mom, Mau × Mys, and Mom × Mys). Notably, in comparison of mating propensities of male or female strains, males from the KM strain of *D. albomicans* and the Mys strain of *D. nasuta* were superior in the mating competitions. In contrast, males from the Mau strain of *D. nasuta* were inferior and defeated in all multiple-choice tests. Females from the Cam strain of *D. nasuta* were most receptive. Both Mau males and females seemed to be inferior to mates from the Cam strain when they were tested together, and this test showed the strongest (but only marginally significant) sexual isolation among all combinations.

In the 16 interspecific combinations strains of the two species did mate randomly with each other. None of sexual isolation indices showed a significant deviation from random mating. Females of both species did not show differences in mating propensity except in one combination (MYH × Mom), where MYH strain females were more receptive than Mom females. In contrast, differences in male mating propensities between these two species were detected in seven combinations (KM × Mau, IR × Mau, SHL × Mau, MYH × Cam, KM × Cam, KM × Mom, and MYH × Mys). Interestingly, the Mau males were also significantly inferior in the interspecific mating competitions, whereas the KM males were superior in the competitions, corroborating the finding in the intraspecific combinations. When the KM and Mys males that were superior in the intraspecific competitions were allowed to compete for mating directly in one interspecific combination, they did not show a significant difference in acquiring mates. Overall, there was no evidence to show any sexual isolation between these two species, even though variance of male mating propensity is larger than that of female mating propensity.

### Courtship

#### Intraspecific combinations

**Figure 1:**
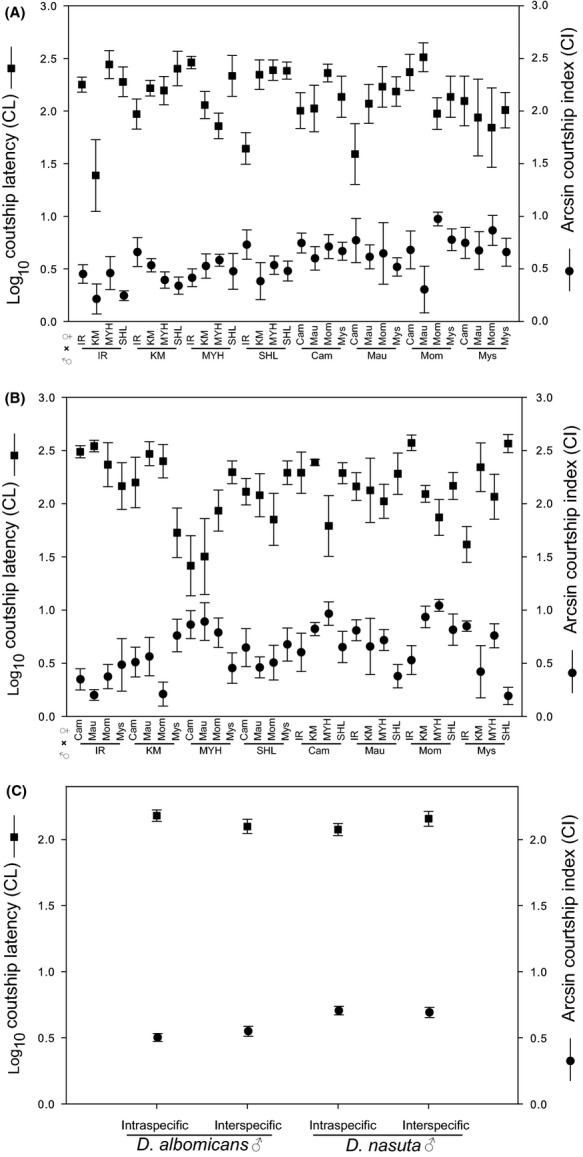
Courtship latency (CL, left *Y* axis) and courtship index (CI, right *Y* axis) of males in intra and interspecific pairwise trials 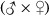 among four strains from each of the two species *Drosophila albomicans* and *D. nasuta*. (A) CL and CI in the 32 intraspecific combinations. (B) CL and CI in interspecific combinations. (C) Average of CL and CI for each species with regard to intraspecific and interspecific combinations. CL is Log_10_ and CI is Arcsine transformed. Error bar represents 1 × SEM. See Methods for the strain name abbreviations.

We observed courtship behavior using four strains from each of *D. nasuta* and *D. albomicans*, and we measured CL, CI, and CPL within and between species. CL represents time elapsed before a male displays his first courtship toward a female after they are placed together in a chamber. Figure 1A shows the CLs of males in 16 intraspecific pairings from each of two species, whereas Figure 1C presents the summary statistics (overall mean and standard error) of CLs and CIs for each species. We performed a three-way nested ANOVA on species, male strain, and female strain (both nested within species) as independent factors (Table 2a). Males of these two species did not show differences in latency to court their conspecific females (*P* < 0.2602; Fig. 1C), but their CLs were significantly influenced by their interaction with females (*P* < 0.0014). To further gauge the nature of this significant interaction between sexes, we next performed a two-way ANOVA on male strain and female strain as independent factors for each species separately. All male strains from both species did not contribute to differences in the CLs (*D. albomicans*, *P* < 0.6709; *D. nasuta*, *P* < 0.8161), neither did the *D. nasuta* female strains. However, the *D. albomicans* female strains and an interaction between sexes did have significant influence on the CLs (*P* < 0.0142 and *P* < 0.0001, respectively), indicating that there might be some genetic variance within *D. albomicans* with regards to female ability to elicit male courtship behavior.

**Table 2:** Summary of ANOVAs for courtship latency (CL) and courtship index (CI) in the intra and interspecific combinations

	Factor	df	SS	MS	*F*-value	*P*
(a) Intraspecific: CL	Species	1	35.7	35.677	1.2753	0.2602
	Species (male strain)	6	66.4	11.071	0.3958	0.8812
	Species (female strain)	6	247.4	41.228	1.4737	0.1891
	Species (male strain × female strain)	18	1228.7	68.259	2.4400	0.0014**
(b) Intraspecific: CI	Species	1	2.3828	2.3828	24.7011	0.0001***
	Species (male strain)	6	0.4271	0.0712	0.7379	0.6197
	Species (female strain)	6	0.9997	0.1666	1.7273	0.1165
	Species (male strain × female strain)	18	2.4336	0.1352	1.4016	0.1342
(c) Interspecific: CL	Species	1	3.97	3.97	0.1517	0.6976
	Species (male strain)	6	453.86	75.643	2.8908	0.0116*
	Species (female strain)	6	273.13	45.521	1.7397	0.1179
	Species (male strain × female strain)	18	1112.82	61.823	2.3627	0.0032**
(d) Interspecific: CI	Species	1	0.7539	0.7539	8.0405	0.0054**
	Species (male strain)	6	2.3351	0.3892	4.1508	0.0008***
	Species (female strain)	6	1.4593	0.2432	2.5940	0.0214*
	Species (male strain × female strain)	18	3.1757	0.1764	1.8818	0.0239*

* *P* < 0.05;

** *P* < 0.01;

*** *P* < 0.001.

The CI represents the proportion of time a male displays courtship toward a female during the observation period. Both male mating propensity and female receptivity can presumably influence the index (Casares et al. 1998). Similar analyses to CLs were applied to CIs as presented in Figure 1A. A three-way nested ANOVA was performed on the CIs with species, male strains, and female strains as independent factors. CIs differed significantly between the two species, whereas the male and female strains and the interactions between them did not contribute to the CI variances (*P* < 0.0001; Table 2b, Fig. 1C). A further two-way ANOVA on male strain and female strain as independent factors revealed no contribution to CIs from either male or female strains from either of the two species (*P* < 0.8423 for *D. albomicans*; *P* < 0.3714 for *D. nasuta*), suggesting that there is little genetic variance for CIs within these two species. However, we did detect a significant interaction between male and female strains within *D. albomicans* with regards to CIs (*P* < 0.0186), indicating once again that there might be genetic variance within this species with regards to the female's ability to influence the male's courtship behavior.

#### Interspecific combinations

Similar experiments for measuring CLs for the 32 interspecific pairings were performed using the 4 strains of each species (Fig. 1B). A three-way nested ANOVA showed that the latency to court heterospecific females was not significantly different between the two species (*P* < 0.6976; Table 2c, Fig. 1C). The variance of CLs was, however, influenced by both male strains and their interaction with female strains (*P* < 0.0116; *P* < 0.0032, respectively). With a two-way ANOVA separately on male strains of each species and their interaction with heterospecific female strains, we found that CLs of *D. albomicans* males were significantly influenced by male strains of *D. albomicans* (*P* < 0.0024) and their interaction with *D. nasuta* females (*P* < 0.0287). Similarly, the CLs of *D. nasuta* were significantly influenced by *D. albomicans* females (*P* < 0.0230) as well as by their interaction with *D. nasuta* males (*P* < 0.0188). These observations are consistent with the intraspecific combinations where genetic variance of CLs was detected within *D. albomicans*, although here we found some suggestive evidence that *D. nasuta* might also possess genetic variance on male's CLs.

The CIs of the males in the 32 interspecific pairings are presented in Figure 1B as well. Again, a three-way nested ANOVA showed significant differences in the CIs between these two species (*P* < 0.0054; Table 2d, Fig. 1C). The CIs were affected by male strains, female strains, as well as their interactions (*P* < 0.0008; *P* < 0.0214; *P* < 0.0239, respectively). Further analyses by a two-way ANOVA showed that the CIs among the four male strains for each species were significantly different (*P* < 0.0065 for *D. albomicans*; *P* < 0.0157 for *D. nasuta*). The CIs of *D. nasuta* males were influenced by *D. albomicans* females and their interactions (*P* < 0.0022; *P* < 0.0425, respectively). Compared to and combined with intraspecific combinations analyzed above, we can interpret these observations as follows: first, there is an intrinsic genetic difference with regards to the CIs between these two species; second, the CIs are controlled by both courting males and courted females; and third, the variance of CIs within males can be augmented when they are courting heterospecific females.

We can also compare the average CLs and CIs between the intra- and interspecific combinations for each species (Fig. 1C). By a one-way ANOVA of CL and CI on combination, each species did not show a difference in the average CLs between intraspecific and interspecific combinations (*P* < 0.2248 for *D. albomicans*; *P* < 0.2793 for *D. nasuta*), nor in the average CIs (*P* < 0.2768 for *D. albomicans*; *P* < 0.8165 for *D. nasuta*), suggesting that males in both species did not discriminate conspecific females from heterospecific ones, even though we uncovered some influences of females on males’ courtship in the previous analyses.

Finally, we analyzed the CPL data to detect any divergence between species in terms of this metric. CPL represents the time elapsed until a male and a female mate after they are introduced into a chamber and both sexes contribute to the CPL. Therefore, a three-way ANOVA on combination, male strain and female strain as factors was performed. There were no significant differences in the CPLs between the intra- and interspecific combinations (*P* < 0.0811). Furthermore, there were no significant differences in the CPLs among male strains as well as among female strains, respectively (*P* < 0.3830; *P* < 0.1371), indicating that these two species show no significant difference in the CPLs.

Taken together, significantly different CIs were observed between *D. albomicans* and *D. nasuta*. Males of both species, however, did not show differences in courting between conspecific and heterospecific females. Females of both species did not discriminate between conspecific and heterospecific males either. In the end, matings happen randomly between these two species.

### Quantitative variations in CHCs

**Figure 2:**
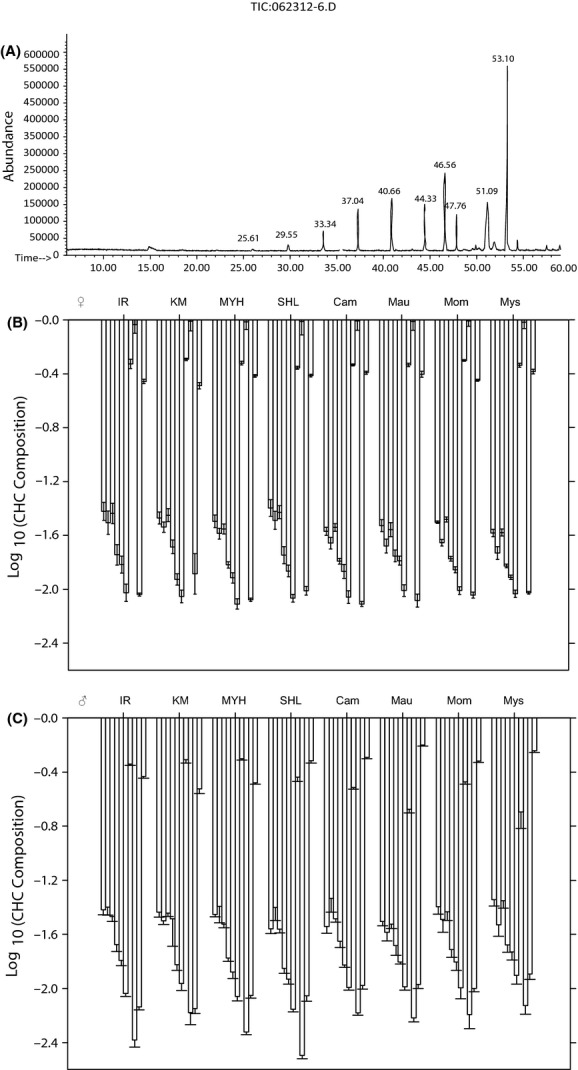
Cuticular hydrocarbon (CHC) variations in the two species *Drosophila albomicans* and *D. nasuta*. (A) Typical gas chromatograph with 10 CHCs identified in this study. The percentages and their Log_10_ transformations of each of the 10 CHCs (from columns 1 to 10 from left to right in each sample) are summarized (Mean ± SEM) for females (B) and males (C). See Methods for the strain name abbreviations.

Male and females from each of both species, with five replicates, were analyzed for CHC profiles. Ten CHCs were identified from the chromatograph, all present in both sexes of these two species (Fig. 2A and Table 3). There are two major CHCs, 2-methyl octacosane (#7) and 2-methyl triacontane (#10), and eight minor ones of alkanes. Their chromatographic peak areas were quantified and normalized so the sum of all 10 proportional values was 1. The proportions of these CHCs for all 8 strains used were log_10_ transformed and presented separately for females (Fig. 2B) and males (Fig. 2C). Here, we found no qualitative differences between species or sexes, but there were quantitative differences.

**Table 3:** Gas chromatographic peaks of CHCs of *Drosophila albomicans* and *D. nasuta*, and their loadings on the first three principal

Peak	Retention time	Hydrocarbons	Formula	MW	Principal component		

					1	2	3
					Eigenvalue		

					4.3065	2.0002	1.2783
1	25.61	n-tricosane	C_23_H_48_	324	**0.748**	0.231	**0.422**
2	29.55	n-tetracosane	C_24_H_50_	338	**0.379**	0.106	**0.733**
3	33.34	n-pentacosane	C_25_H_52_	352	**0.819**	0.170	**0.340**
4	37.04	n-hexacosane	C_27_H_54_	366	**0.686**	0.297	−0.214
5	40.66	n-heptacosane	C_27_H_56_	380	**0.878**	0.020	−0.157
6	44.33	n-octacosane	C_28_H_58_	394	**0.861**	0.007	−**0.306**
7	46.56	2-methyl octacosane	C_29_H_60_	408	−**0.601**	**0.725**	−0.043
8	47.76	n-nonacosane	C_29_H_60_	408	**0.745**	0.068	−**0.472**
9	51.09	n-triacontane	C_30_H_62_	422	0.055	−**0.674**	0.234
10	53.10	2-methyl triacontane	C_31_H_64_	436	0.174	−**0.911**	−0.062

CHCs that contribute significantly to the three principal components are in bold.

A PC analysis was performed to reduce the dimensionality of the original CHC observations to three major PCs with their eigenvalues >1.0 (Norman and Streiner 2008) and accounting for 76% of the total variance in the CHC compositions. The top three PCs accounted for 43%, 20%, and 13%, respectively. Factor loadings of each CHC on the three major PCs are shown in Table 3. Conventionally, loadings >0.3 are thought to be biologically meaningful (Tabachinick and Fidell 2012). Thus, there are eight CHCs significantly contributing to the PC1, whereas there are three and five CHCs significantly contributing to the PC2 and PC3, respectively. Nested ANOVAs of these three PCs were carried out to detect any significant influences by species, strain, and sex, with the latter two factors nested within species (Table 4). For PC1, it was the sex (*P* < 0.0005), whereas for PC2, all three factors (*P* < 0.0001 for species; *P* < 0.0004 for strain; and *P* < 0.0001 for sex) and for PC3, the species (*P* < 0.0001), which significantly affected the PCs. These results argue that the quantitative compositions of CHCs vary significantly between these two species and across the two sexes and different strains. However, as we mentioned before, there were no unique CHCs detected only within a subset of the fly samples.

**Table 4:** Summary of ANOVAs of the principal components (PCs) by three factors (species, strain, and sex, with the latter two nested within species)

PC	Factor	df	SS	MS	*F*-value	*P*
1	Species	1	10.7808	10.7808	3.0103	0.0871
	Strain (species)	6	18.0867	3.0144	0.8417	0.5421
	Sex (species)	2	60.6589	30.3295	8.4689	0.0005*
2	Species	1	38.9405	38.9405	45.4300	<0.0001*
	Strain (species)	6	24.8336	4.1389	4.8287	0.0004*
	Sex (species)	2	34.2372	17.1186	19.9714	<0.0001*
3	Species	1	20.9041	20.9041	22.0641	<0.0001*
	Strain (species)	6	6.2983	1.0497	1.1080	0.3666
	Sex (species)	2	7.4672	3.7336	3.9408	0.0239*

Asterisk denotes significant *P*-values.

It would be interesting to explore a possible correlation between the CHCs and courtship because CHCs have been evidently documented to play pheromonal roles in *Drosophila* mate recognition and courtship (reviewed in Ferveur 2005). We used the CHC and courtship data obtained from 5 pairs per strain for each species (*N* = 80) in intraspecific combinations, albeit not from the same samples. A simple regression analysis was performed six times between the CLs or CIs and each of the three PCs from females. Only in one regression was the CLs and the PC1 significantly correlated (*P* < 0.0042). When this regression was independently performed for each species, the significance was restricted to *D. nasuta* only (*P* < 0.0004; *P* < 0.3362 for *D. albomicans*). This suggests that CHCs might be used as chemical cues by *D. nasuta* males before they initiate the courtship (i.e., CL). Overall then, the CHCs seem to have played minimal roles in the courtships in these two species. This finding is somewhat unexpected because both courtship behaviors (Fig. 1C) and CHC compositions (Table 4) vary across species, as reported in the richly documented cases of pheromonal CHCs (reviewed in Ferveur 2005), yet in these two species the pheromonal function of CHCs seems to be minimal.

## Discussion

We demonstrated in this study that two geographically allopatric species, *D. albomicans* and *D. nasuta*, randomly mated with each other. We found random matings occurring within and between species in all tests except one intraspecific pair that showed some level of sexual isolation, but it was not statistically significant. The fact that *D. albomicans* and *D. nasuta* mate randomly with each other suggests that these two species might have limited divergence in traits related to mate choice. We analyzed variance in courtship and CHCs within and between species. We detected no differences in the CLs, but we did find significant differences in the CIs between these two species. The CIs were influenced by both sexes as well as by interaction between the sexes. Males and females of both species, however, did not discriminate between conspecific and heterospecific partners. We identified 10 CHCs from both species, but these CHCs were only quantitatively, not qualitatively, different between the two species. A PC analysis ended with three PCs that account for 76% of the CHC variance, and all the three factors (species, strain, and sex) significantly influenced the CHC compositions in these two species. Our regression analysis for the CLs and CIs on the PCs suggested that the CHCs in these species played a negligible role in courtship behavior and did not give rise to sexual isolation between these two species. We conclude that the two *Drosophila* species, *D. albomicans* and *D. nasuta*, have little divergence of sexual behavior and no sexual isolation between them, and they appear to be only at a very incipient stage of speciation.

Driven by sexual selection, courtship is often species specific and the behavioral divergence within species often serves to bolster reproductive isolation between species (Spiess 1987). Comparisons of species-specific behavior among closely related species clearly documented that courtship behaviors sexually stimulate mating partners within species on one hand, and impede mating between species on the other hand (reviewed in Markow and O'Grady 2005). The courtship and mating behavior of the *D. nasuta* subgroup species have been studied previously (Spieth 1969; Lambert 1982): males of this subgroup commonly display a basic mating pattern, but quantitative differences were observed between individual males within species. When a male and a female are placed in a mating chamber in the laboratory, the male displays orientation, tapping, following, circling, vibration, and attempted copulation, and the female readily mates with a conspecific male. Mating occurs after a male displays a courtship, and the duration of copulation is relatively long, ∼25 min, compared with other species such as *D. pseudoobscura* (Y.-K. Kim, pers. obs.). Our courtship analysis between *D. albomicans* and *D. nasuta* has demonstrated that these two species are quantitatively different in the CIs and the strains within species also show differences in the CIs. This observation seems to support an earlier suggestion that there is an evolutionary continuum from genetic variance of sexual behaviors within species to sexual isolation between species (Carson 2003).

During courtship, a male exchanges various signals with a prospective female mate: acoustic, chemical, and visual (reviewed in Ehrman and Kim 1997; Greenspan and Ferveur 2000; Lasbleiz et al. 2006). The relative importance of these sensory modalities varies with species. Among chemical signals, CHCs represent an important part of the specific mate recognition system and are crucial in *Drosophila* mate recognition (reviewed in Ferveur 2005). Several studies have demonstrated that CHCs contribute to sexual isolation between geographic strains or between closely related species at the incipient stage of speciation. For instance, 7,11-heptacosadiene (7,11-HD) and 7-tricosene (7-T) are predominant in females of *D. melanogaster* and *D. simulans*, respectively, and the difference in the CHC profiles causes sexual isolation between the two species (Coyne 1996). Similarly, sexual isolation between Zimbabwe and cosmopolitan races of *D. melanogaster* is correlated with differences in 7,11-HD and 5,9-HD (Coyne et al. 1999), and a *desaturase*-2 (*desat*-2) pheromone locus is responsible for polymorphism in the female CHCs (Dallerac et al. 2000; Fang et al. 2002; Greenberg et al. 2003; Coyne and Elwyn 2006). In addition, an inhibitory male pheromone, 7-T, is also correlated with the Zimbabwe females’ discrimination against the cosmopolitan males (Grillet et al. 2012). Recently, a partial sexual isolation between US and Caribbean populations of *D. melanogaster*, but random mating between West African and Caribbean populations, was reported, showing that the *desat*-2 locus is responsible for climatic adaptation and sexual isolation across the US–Caribbean region (Yukilevich and True 2008,b). Furthermore, CHCs have been differentiated in *D. serrata* strains sympatric with *D. birchii* as a result of reproductive character displacement (Higgie et al. 2000). In this study, we found no qualitative divergence of CHCs in *D. albomicans* and *D. nasuta*, but we did find significant quantitative variations in the CHCs across species, sex, and strains (Fig. 2B and C; Table 4). This observation suggests that there is a genetic basis for these variations although all 8 strains used in this study have been maintained in the laboratory for at least 20 years. Both *D. albomicans* and *D. nasuta* show broad geographic distributions and there might be more differences in the CHC profiles among natural populations of both species. For example, *D. melanogaster* males that range from temperate to equatorial geographic areas show a huge amount of variation in the ratio of two major hydrocarbons, 7-tricosene and 7-pentacosene (Rouault et al. 2000). Cobb et al. (1990) demonstrated that the CHC compositions between ancestral and derived populations of *D. sechellia* changed after the former was reared on a special food, the latter on a standard *Drosophila* food in the laboratory. Little studies on the ecological differentiation among natural populations of both *D. albomicans* and *D. nasuta* had been performed. We have shown in this study that the CHCs play a negligible role in eliciting sexual isolation between *D. albomicans* and *D. nasuta*. It is unlikely, though, that the lack of sexual isolation between these two species is a result of their convergent adaptation to the same laboratory environment (Houot et al. 2010). In terms of acoustic signals, we did not study roles of courtship song in sexual isolation between these species, but courtship song might be a good candidate for our future studies. Cobb et al. (1990) reported differences in courtship song although they did not find significant sexual isolation between ancestral and derived populations of *D. sechellia* in the laboratory.

Quantification of sexual isolation between *D. albomicans* and *D. nasuta* is affected by factors including observation methods, statistical analyses, strains used, and environmental conditions. A few studies have been performed to measure sexual isolation between these two species (Kitagawa et al. 1982; Ramachandra and Ranganath 1987; Tanuja et al. 2001a,b; Chang and Tai 2007), but the conclusions differed among these studies. Using male-choice tests, Kitagawa et al. (1982) reported random matings between interspecific populations of *D. albomicans* and *D. nasuta*. Using no-, female-, male- and multiple-choice tests, Ramachandra and his colleagues showed a significant departure from random mating between these two species (Ramachandra and Ranganath 1987; Tanuja et al. 2001a,b). Chang and Tai (2007) reported asymmetric sexual isolation between the two species with more homogamic matings occurring in *D. albomicans* than in *D. nasuta*. These discrepancies might be caused by the different methods used, some being flawed. For example, Kitagawa et al. (1982) used male choice tests and placed 15 males as well as 10 conspecific and 10 heterospecific females in a mating chamber and observed matings until either 10 matings occurred or 1 h passed. In the Ranganath studies (Ramachandra and Ranganath 1987; Tanuja et al. 2001a,b), a smaller number of flies, that is, one male and two females or two pairs of flies, were put in a mating chamber and observed for 5 h in each trial, and the observation was repeated multiple times for thousands of matings. In the last study (Chang and Tai 2007), three flies were placed in a small chamber for both female and male choice tests and observed until mating occurred. They also scored mating events among 400 flies in a single chamber for 1.5 h, with 100 of each sex from these two species in the multiple-choice situations. During observation, the mated flies were removed from the chamber, but this presumably disturbed the potential mating of other flies in the same mating chamber. Such methodological variations will certainly contribute to discrepancies in the conclusions.

Related to the observational methods, various isolation indices were used to measure sexual isolation in these studies. As pointed out by Rolán-Alvarez and Caballero (2000) and Pérez-Figueroa et al. (2005), these indices may actually measure subtly different things. They particularly criticized the *χ*^2^ and joint isolation statistics for having the largest variances of asymptotic biases due to uncorrected marginal effect, and for being more affected by sample sizes than any other statistics. However, the biases are drastically reduced in studies with big sample sizes, for example, in Kim et al. (2012) and this study as well, where *χ*^2^ and *I*_PSI_ are similar. Last but not least, different strains, temperatures, illumination, and humidity in different laboratories certainly contribute to the discrepancies in these studies. Thus, we need to carefully consider which methods are appropriate to the mating observations in *Drosophila*. In many *Drosophila* species, multiple males and females, not hundreds of flies, are randomly distributed near food resources where interactions between potential mating partners occur (Spieth and Ringo 1983). We assume that the multiple-choice tests carried out in this study are more like those in natural environments of the two subject species.

*Drosophila nasuta* and *D. albomicans* have been used to study postzygotic reproductive isolation such as hybrid sterility or breakdown in the early stage of speciation between two species. Both F_1_ interspecific hybrid males and females are apparently fertile (Kitagawa et al. 1982), but some of the F_2_ hybrid males are sterile (Ranganath and Krishnamurthy 1981), presumably because they had the XO genotype as a consequence of losing the Y chromosomes during meiosis in their father (Chang and Kung 2008). An abnormal sex ratio was detected in both interspecific and intraspecific crosses. When the Japanese *D. albomicans* females are crossed to *D. nasuta* males, a striking excess of females are observed in F_2_ generation. Crosses between some geographically separated strains of *D. albomicans* also show female-biased sex ratio distortion, but all crosses between *D. nasuta* strains are normal (Inoue and Kitagawa 1990). The evidence is strong for a suppressed meiotic drive system in *D. albomicans*, and significant evolutionary consequences would presumably have been left behind along the rise and fall of the meiotic drive system in this species (Meiklejohn and Tao 2010). Meiotic drive is just one of many intragenomic conflicts discovered to date (Burt and Trivers 2006). Meiklejohn and Tao (2010) argued that epigenetic regulation of the sex chromosomes, as well as postzygotic incompatibilities (hybrid sterility), could be among these consequences. Another possible consequence is selection for more promiscuous females (Price et al. 2008), but it is not supported by our study.

In a parallel study (our unpublished data), we have observed a high frequency of sex chromosome nondisjunction in the F_1_ hybrid males between *D. albomicans* and *D. nasuta*, in addition to sex ratio meiotic drive. The F_1_ hybrid males also have severely reduced fertility. The evidence suggests hybrid incompatibility in the germline of the F_1_ male, in contrast to the lack of premating sexual isolation between *D. albomicans* and *D. nasuta* found in this study. We conclude that the evolution of postzygotic isolation precedes that of prezygotic isolation, and intragenomic conflict might be a major evolutionary mechanism for the faster evolution of postzygotic isolation, at least in the case of *D. albomicans* and *D. nasuta*.
